# Spongia Tosta

**Published:** 1887-12

**Authors:** C. Carleton Smith


### "SPONGIA TOSTA."

C. Carleton Smith, M. D.

The simple toasting of a piece of sponge seems to have been an inspiration when we consider how many lives have been saved by this remedial agent of patients suffering with acute laryngitis and croup, both alarming diseases. This drug, as we well know, contains Bromine, Iodine, and perhaps some calcareous matter.

Dr. Hering taught us that it is particularly well adapted to light-complexioned patients, while Iodine has a greater affinity for persons who have dark complexions and black eyes. From what we glean from the various provings of this drug, it acts with great intensity upon the glandular system and mucous surfaces. Hence its great use in goitre. In cases that come to us of this character we will find, as one of the leading conditions indicative of this drug, suffocating spells at night, while the gland is very hard to the feel, and enlarges rapidly.

This sense of suffocation is a subjective symptom belonging to the diseased condition, and is not necessarily caused by the enlargement of the involved gland, for with comparatively small goitres we find the same symptom quite prominent, and is always an indication that our patient requires Spongia.

We find this drug acts specifically upon the testicles, causing great enlargement and intense hardness of these glands.

Hence we think of Spongia in badly treated cases of orchitis, and also in inflammation of an acute form arising from the suppression of gonorrhœal discharges by injections. The patient complains of a jamming, crowding pain also, and, what is remarkable, a *choking* feeling is often present, especially in orchitis. Allied to Spongia in this latter condition, we have Pulsatilla and Merc. solub., and as both these last-named drugs have greenish-yellow discharge of what little secretion may be left, we distinguish them in this way : Puls. has drawing, tensive pains through cords into abdomen, enlarged prostate, and stools flattened out and small in size. Under Merc. the testicles are so hard they shine, and are also sweaty, and the yellowish-green discharge is especially troublesome during the night. We are all well acquainted with the wonderful curative action of Spongia in acute laryngitis, a diseased condition which is at times most formidable. We have here a harsh, barking cough, with the larynx exceedingly sensitive to the touch or contact of clothing. This latter condition is exactly similar to Lachesis, a drug that is often given in mistake, on account of this similarity, in place of Spongia. In such cases when Spongia is indicated the patient cannot even turn the head without the effort brings on a suffocative attack. Sambucus may be considered here in this connection as being especially indicated when these spasms of the larynx occur frequently without regard to any effort for motion being made by the patient. Spongia has won its chief laurels in croup, the indications being as follows: Breathing anxious, worse during inhalation, which distinguishes it from Acon., which is worse during exhalation. Wheezing is the great characteristic for Spongia, and patient cannot lie down on account of increase of dyspnœa, but must sit up and lean forward. Should he sleep in this position he invariably wakes up with suffocative spells. (Lachesis.)

Spongia croup generally comes on in the evening or night. Hepar croup, in or toward morning, and aggravation of symptoms from juice of orange.

Spongia has, according to its proving, a decided action upon the lung tissue. Hence we find it invaluable in cases of phthisis pulmonalis. It has, like Ant. tart., solidification of these organs, and hard, *ringing*, *metallic* cough, which latter is peculiar to itself. Deep breathing, talking, and inhaling dry, cold air causes decided aggravation of this cough. The act of eating or drinking ameliorates the cough, a condition we find also under Anacardium. Patients who need Spongia in tuberculous disease complain a great deal of sudden weakness overtaking them when walking abroad.

This seems to be due to a congestive condition which this drug has the faculty of causing. The books teach us that Hepar must follow Spongia when we find the Spongia cough continues, but with more rattling of mucus. But this I consider bad advice, for if a patient is improving under Spongia, that improvement is signaled by a change to a moist rattling, and then Spongia ought to be stopped, if it had been given in repeated doses, and a cure will follow—the Spongia patient *wheezes*, and when this wheezing is changed to loose mucous cough the patient is getting well. In cases of phthisis Spongia must be thought of when the sufferers have sudden and oft-repeated flushes of heat, similar to Xanthoxylum, only the former has aggravation when thinking of them.

Spongia patients complain a great deal of chills which run across the back, and even shake while hugging a hot stove: these flushes never seem to affect the thighs—they being chilly and even numb.

In organic affections of the heart, we could not well get along without this valuable remedy. We have here great dyspnœa, the patient wholly unable to lie flat on the back without bringing on at once terrible suffocation.

Hence, you will usually find these cases sitting up in bed leaning a little forward, and their faces wearing a most anxious expression, cheeks flushed, and breathing rapid. Aconite in these cases follows Spongia well, and the former has also waking up from sleep with great distress, the face quite red.

Young ladies who from over-indulgence in dancing suddenly become faint and sink down helplessly, with short, difficult breathing, are quickly relieved by Spongia—a single dose.

Spongia has hoarseness, with difficulty of breathing, as if a *cork* were sticking in the larynx, while Bromine has feeling as though the patient had to breathe through a *sponge*. In its action on the general functions in women, we find a symptom exactly similar to Calcarea, viz.: too early and too profuse menstruation. But under the former remedy, the patient has severe backache just before the menstrual flow, which is soon followed by palpitation of the heart, after which phenomena the flow begins.

Spongia is a most powerful antipsoric, and therefore should not be frequently repeated, even in acute diseases.

Dr. Lippe remarked once in my presence that it is unsafe to rapidly repeat this drug in membranous croup when indicated, and that many cases are spoiled by so doing, and, worse than this, the patients' lives were placed in jeopardy. Dr. Lippe follows the excellent plan of giving one dose *high* and awaiting its action.

The mental symptoms are worthy of note, they being somewhat similar to Puls., in so far that the patient is constantly weeping and quite inconsolable. She would rather die on the spot than suffer as she does. But, unlike Puls., she becomes extremely vehement at times, scolding fearfully. Another and very important symptom of the mind is, she is constantly expressing a fear that she will certainly die of suffocation. Also is possessed with the idea that her head is being blown up like an elastic balloon. And one of the most peculiar symptoms elicited in a number of provers was "a constant desire to sing."

Smoking tobacco and also inhaling the smoke always brings on the Spongia cough. Spongia follows Veratrum-alb. and Arsenicum well in cases of angina pectoris. It also supports Stannum nicely where this drug is indicated in a given case.

Hepar always *follows* Spongia, does not precede it, and is very similar to this drug in severe colds. Calcarea carb. frequently *follows* Spongia. And after Spongia in acute attacks of hoarseness Carbo veg. is often invaluable.

The most perfect antidote is Camphor.

